# Items in Brief

**Published:** 1878-08

**Authors:** 


### Items in Brief.

—The announcement of the Massachusettes Medical Society's prize for next year has been made by Dr. Goss. The first of these annual prizes has been carried off by Dr. Thomas Dwight, who presented a paper entitled The Indentification of the Human Skeleton. We understand that several essays of exceptional merit competed for the prize. The following are the terms upon which the competition is based:—

The committee on publications are authorized to offer the sum of *two hundred dollars* as a prize, or honorarium, to any one Fellow of the society who shall give to the satisfaction of said committee, on or before the 15th of April next, in an essay or report (worthy of a prize), the best and fullest evidence of any original or meritorious *professional work,* done by himself during the two years next preceeding said date, in experimental investigations, scientific researches, or clinical observations.

Papers may be sent to Dr George C. Shattuck, 6 Newbury Street, Boston, on or before April 15, 1869, with motto and name, as usual in such cases.

—M. Galeyowski presented at a session of the Society of Biology at Paris, in March, a new mydriatic—*Duboi ia myopsorida,* a plant belonging to the scrophulariaceæ, from Australia. It is said to be stronger and more lasting in its effect upon the pupil than atropia, and is without undesirable constitutional effect.—

—H. W. Williams, M. D., Professor of Ophthalmology in Harvard Medical College, says that one drop of a solution of two grains of sulphate of eserine in an ounce of water, instilled beneath the eyelids begins to contract the pupil within fifteen minutes, and produce spasm of the accomodation which often reaches a high degree in a few minutes, and there is often twitching of the eyelids and sometimes empra-orbital pain, usually slight. These symptoms are usually at their height within an hour, after which they diminish, and at the end of the second hour have in most cases disappeared, with the exception of the contraction of the pupil, which persists for eight hours or longer. In his experiments with chloe-hydrate of pilocarpine, the active principle of jaborandi, an equivalent degree of contraction of pupil and myopia was produced with less accommodative spasm, supre-orbital pain and conjunctival irritation. He considers these agents as unquestionably capable of great service, has used them in perforating ulcer of the cornea, commends their use in paralysis of accommodation and mydriosis, in glaucoma and sympathetic ophthalmia in connection with other surgical means. In iritis they would be harmful. Prof. Nalt in his work on Diseases and Injuries of the Eye says that Calabar Bean has not been useful in his hands and is not to be relied on to draw in a prolapse of the iris after cataract and other operations. The internal dose of powdered Calabar Bean (*phyrostigma*) is one to three grains, of the extract. one-eighth to one-sixth of a grain in tetanus. The sulphate of eserine is ten to fifteen times more powerful than the extract.

—Carl Bartels, Professor and Director of the Medical Clinic at the University of Kiel, died on the twentieth of June, at the age of fifty-five. He was the author of the excellent contribution to Ziemssen's Cyclopœdia on Structural Diseases of the Kidney.

